# Preparation and Preliminary Dielectric Characterization of Structured C_60_-Thiol-Ene Polymer Nanocomposites Assembled Using the Thiol-Ene Click Reaction

**DOI:** 10.3390/ma8115424

**Published:** 2015-11-18

**Authors:** Hanaa M. Ahmed, Amber D. Windham, Maryam M. Al-Ejji, Noora H. Al-Qahtani, Mohammad K. Hassan, Kenneth A. Mauritz, Randy K. Buchanan, J. Paige Buchanan

**Affiliations:** 1 Department of Chemistry and Biochemistry, University of Southern Mississippi, 118 College Drive, Hattiesburg, MS 39406, USA; Hanaa.ahmed76@gmail.com (H.M.A.); amber.gresham@eagles.usm.edu (A.D.W.); 2 Center for Advanced Materials, Qatar University, Doha P.O. Box 2713, Qatar; maryam.alejji@qu.edu.qa (M.M.A.-E.); noora.alqahtani@qu.edu.qa (N.H.A.-Q.); mohamed.hassan@qu.edu.qa (M.K.H.); 3 School of Polymers and High Performance Materials, University of Southern Mississippi, Hattiesburg, MS 39406, USA; kenneth.mauritz@usm.edu; 4 U.S. Army Engineer Research and Development Center, Information Technology Laboratory, Institute for Systems Engineering Research, 3909 Halls Ferry Road, Vicksburg, MS 39180, USA; Randy.K.Buchanan@erdc.dren.mil; 5 Faculty of Engineering, Benha University, Shoubra, Benha 13 512, Egypt

**Keywords:** dielectric properties, fullerenol, thiol-ene, nanocomposites

## Abstract

Fullerene-containing materials have the ability to store and release electrical energy. Therefore, fullerenes may ultimately find use in high-voltage equipment devices or as super capacitors for high electric energy storage due to this ease of manipulating their excellent dielectric properties and their high volume resistivity. A series of structured fullerene (C_60_) polymer nanocomposites were assembled using the thiol-ene click reaction, between alkyl thiols and allyl functionalized C_60_ derivatives. The resulting high-density C_60_-urethane-thiol-ene (C_60_-Thiol-Ene) networks possessed excellent mechanical properties. These novel networks were characterized using standard techniques, including infrared spectroscopy (FTIR), differential scanning calorimetry (DSC), dynamic mechanical analysis (DMA), and thermal gravimetric analysis (TGA). The dielectric spectra for the prepared samples were determined over a broad frequency range at room temperature using a broadband dielectric spectrometer and a semiconductor characterization system. The changes in thermo-mechanical and electrical properties of these novel fullerene-thiol-ene composite films were measured as a function of the C_60_ content, and samples characterized by high dielectric permittivity and low dielectric loss were produced. In this process, variations in chemical composition of the networks were correlated to performance characteristics.

## 1. Introduction

C_60_ fullerene-containing polymers are receiving increased attention due to their remarkable properties and anticipated applications. Recent reviews describe the inclusion of C_60_ into main-chain, side-chain, cross-linked, and star-shaped polymer configurations [1,2]. C_60_’s three dimensional structure yields a unique versatility in constructing high-molecular weight polymer architectures. An interesting feature of these C_60_-polymer nanocomposites lies in the possibility of tuning the physical properties and therefore resulting potential applications of the composite through modification of the chemical linkages among the matrix constituents. Demonstrating this versatility, there are interesting reports of C_60_’s incorporation into polymer composites as blends to create photo-active and stimuli-responsive coatings [3], C_60_-derivative-styrene blends [4] and covalent C_60_-styrene copolymers [5], C_60_-polyurethanes via reaction of the hydroxylated C_60_ [6,7], and composites prepared in polyethylene and polyamide matrix materials [8,9].

In addition to the standard techniques to measure thermal and mechanical performance of polymer composite materials, the lesser-used method of dielectric spectroscopy is employed to characterize molecular dynamics and electrical polarizability of the networks [10,11]. The dielectric response results from the interaction of dipoles or polarizable elements with an oscillating applied electric field (*f*) at a given temperature. The essential quantity is the complex dielectric permittivity which is given by Equation (1),

(1) $$\text{ε}\;*\;\left(\text{ω}\right)\;=\;{\text{ε}}^{\prime }\;\left(\text{ω}\right)\;-\;i{\text{ε}}^{\prime \prime }\;\left(\text{ω}\right)$$

where ω is the angular frequency = 2π*f* and *i* = √−1. εʹ is referred to as the real permittivity and describes the material polarizability due to dipole reorientation, deformation of delocalized electron distributions, or interfacial polarization internal to the sample or at the sample/electrode interface. εʹʹ, the imaginary, or loss permittivity, is proportional to the energy dissipated per cycle during any of these processes, termed “relaxations”. Modern broadband dielectric spectroscopy (BDS) techniques allow the analysis of samples across a wide frequency window (10^−3^ to 10^6^ Hz) and is therefore a very powerful tool for probing molecular dynamics of polymers *versus* temperature [10].

Recently our group reported the preparation and characterization of a series of fullerene (C_60_ and Sc_3_N@C_80_) polymer nanocomposites built up from a hydroxylated fullerene core, which was crosslinked with a diisocyanate elastomer polyether-based oligomer [7]. Multiple relaxations were characterized from the dielectric analysis of this system and assigned to the glass transition temperature (T_g_), crankshaft motions of the ether segments, reorientation of the hydroxylated fullerene cages, and other local motions. Overall these fullerene-polymer networks could be rendered quite polarizable, but were complicated by a rather challenging synthetic strategy. Most recently our group has focused on efforts to increase the loading capacity of fullerene-polymer dielectric networks which, in turn, is expected to increase the dielectric permittivity while maintaining the desired low dielectric loss. The incorporation of C_60_ and other fullerenes is notoriously hampered by the poor processing of fullerenes. Using our hydroxylated C_60_ fullerene as the core structure, we have successfully implemented the “thiol-ene” click reaction to prepare a unique series of nanocomposites of high C_60_ loading. Herein we report the synthesis and essential characterization of C_60_-urethane-thiol-ene (C_60_-Thiol-Ene) networks, thermal stability, mechanical, and dielectric properties.

## 2. Results and Discussion

**Scheme 1:**
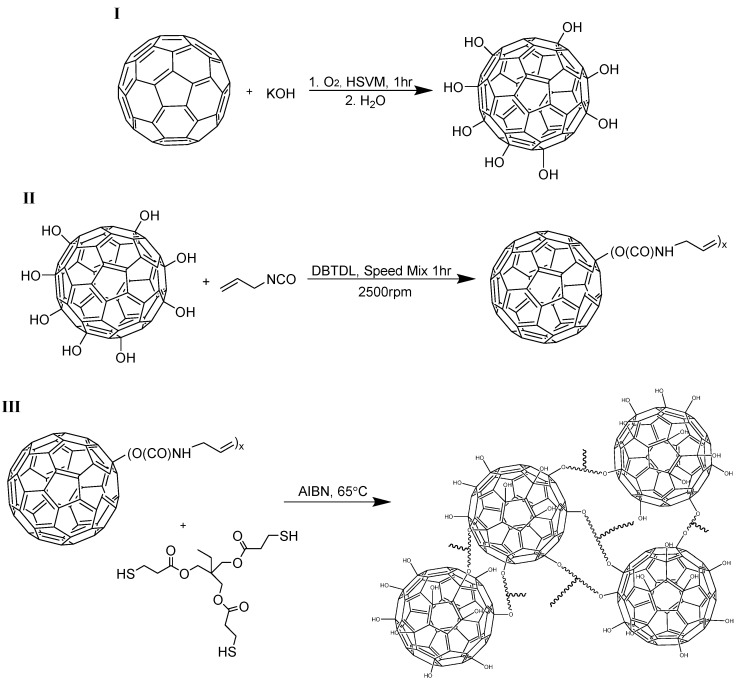
Preparation of C_60_-Thiol-Ene Network.

The general synthesis route for the preparation of the fullerene-polymer networks, described as C_60_-Thiol-Ene, is provided in Scheme 1. In this method, there is a sequential buildup of the network from the reaction of hydroxylated fullerene with allyl isocyanate to produce a reactive “ene” monomer and its subsequent reaction with an alkyl “thiol” in the prototypical thiol-ene reaction. There are a number of excellent reviews of the thiol-ene click reaction [12,13,14]. The thiol-ene reaction is based on a free-radical, step-growth polymerization mechanism. In general, the thiol-ene reaction's selectivity, high monomer conversions, insensitivity to molecular oxygen, and few competing reactions are many of the reasons for the recent popularity. A large number of commercially available thiols and enes permits the tailoring of polymer network properties for a variety of applications, and the attractive polymerization rates and uniform polymer networks produced make the thiol-ene matrix an ideal choice for polymer-particle composites [12,13,15].

The hydroxylation of C_60_ and was performed as previously reported by our group to yield an average of 29 OH groups, C_60_(OH)_29_, with additional characterization provided in supplementary material [7]. Using anhydrous conditions, allyl isocyanate was combined under high shear to C_60_(OH)_29_ in various ratios (2:1, 1:1, and 1:2 functional group equivalents) to yield a series of composites with increasing C_60_ content in the matrix, Table 1. This ene-functionalized C_60_ monomer was combined with the thiol monomer TMPMP and thermal initiator, thereby creating 9, 15, and 27 wt % C_60_ polymer composites, respectively. Following thermal cure brown/black, transparent composites were produced, which were easily removed from the Teflon mold and characterized. Films were sonicated briefly in MeOH to quench any residual isocyanate groups that were not captured by either SH and OH functional groups, particularly important for the 9-C_60_-TE, where an excess of NCO to OH is added initially. At all times a 1:1 stoichiometric ratio of thiol to ene functional groups is maintained. It is noted that there are expected differences in the resulting networks from the combination of monomers chosen, specifically 9-C_60_-TE may have a small fraction of residual urethane, thiourethane, or ene ends. Sample 15-C_60_-TE will represent the ideal network composition from a chemical composition perspective, and 27-C_60_-TE will possess a large fraction of unreacted carbinol groups in the resulting network. However, gel fractions of all samples are considered high, indicating well-formed networks. The contribution of these residuals is considered in drawing conclusions in the characterization of the networks.

**Table 1:** Compositions of prepared C_60_-containing thiol-ene films.

Sample ID	NCO Equiv.	OH Equiv.	SH Equiv.	Wt % C_60_ *
9-C_60_-TE	2	1	2	9
15-C_60_-TE	1	1	1	15
27-C_60_-TE	1	2	1	27

* wt % C_60_ was calculated from the mass of C_60_(OH)_29_ in each sample in relation to the total mass of all nonvolatile film constituents, expressed as a percent.

**Figure 1:**
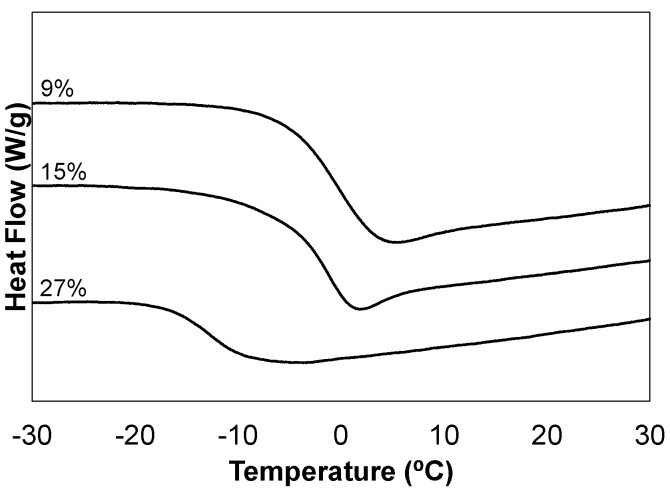
DSC analysis of the C_60_-thiol-ene network film series, C_60_ wt % in composite film noted.

Networks were characterized using common techniques, including gel fraction, infrared spectroscopy (FTIR), thermogravimetric analysis (TGA), differential scanning calorimetry (DSC), and dynamic mechanical analysis (DMA). A summary of the critical values are reported in Table 2. All monomer combinations produced networks characterized by high gel fractions, IR spectrums consistent with the composition, and thermal degradation onset temperatures of approximately 200 °C (IR and TGA plots included in supplementary material). DSC plots are provided in Figure 1, and illustrate the effect of varying the network chemical composition. All samples yield well-defined transitions; however, it is the ideal network 15-C_60_-TE which is most characteristic of thiol-ene networks in appearance. This sample possesses the dip after the thermal step transition which has been attributed to the enthalpic relaxation of the thiol-ene network and has been studied extensively by our group and others [12,16].

**Table 2:** Characterization of C_60_-TE films.

ID	Gel %	T_g_ (°C) by DSC	T_g_ (°C) by DMA	DMA Tan δ Width at ½ Height (°C)	Tan δ Peak Height
9-C_60_-TE	88	−0.7	26.3	31	0.84
15-C_60_-TE	94	−1.1	17.7	26	0.70
27-C_60_-TE	100	−12.9	4.7	19	0.76

**Figure 2:**
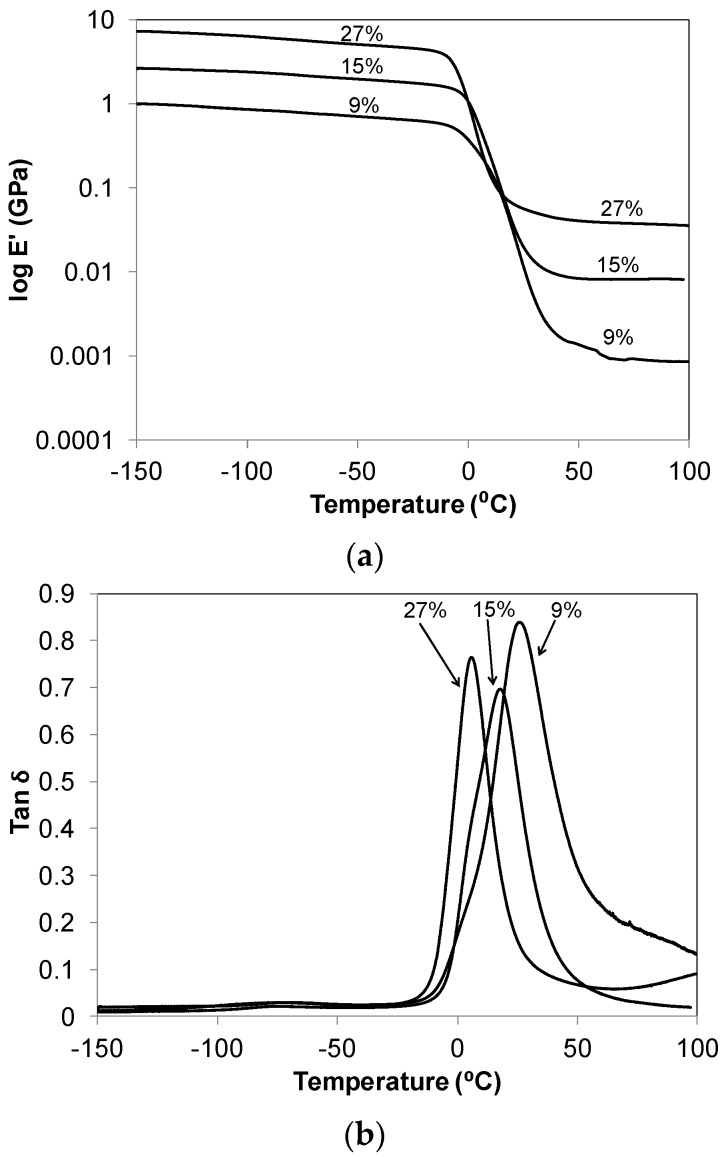
(**a**) Dynamic storage modulus (Eʹ); and (**b**) loss tangent (tan δ) for the C_60_-thiol-ene network film series, C_60_ wt % in composite film noted.

Dynamic storage modulus (E') and loss tangent, tan δ = E"·(loss modulus)/E' *versus* temperature curves for C_60_-thiol-ene networks are provided in Figure 2. The storage modulus curves show a glassy material at low temperatures and a rubbery material at high temperatures with a glass transition at approximately 0 °C that is associated with the onset of long-range chain segmental mobility of the thiol-ene network. The trend in the rubbery state modulus increases in the series as the C_60_ content increases, as expected. This trend for the gel % and the rubbery state modulus also reflect the variation in crosslink density among the samples having different composition. All show a subtle peak in the temperature range of −90 to −50 °C which is associated with the crankshaft motions of the methylene sequences in the soft segments of the thiol-ene networks [7,17,18].

The peak maximum in the tan δ plots, increasing from 26.3 to 4.7 °C, correlates to the increasing C_60_ content of the network and represents the most active region of the glass transition at T_g_. T_g_s obtained by DMA measurements are reported in Table 1 along with those obtained by DSC. Regardless of the analysis method used, the T_g_ decreases systematically with the increase in the C_60_%. This behavior suggests that as the concentration of C_60_(OH)_29_ increases in the network in relationship to the number of fullerene crosslinks, an increase in network free volume, and fullerene mobility results, thus leading to a decrease in the observed Tg. A similar trend was observed by Lu *et al.* [8] in their study of benzylaminofullerene (BAF)-polyethylene composites as a function of the BAF loading. Moreover, the maximum of the tan δ fluctuates, with the ideal network having the lowest maximum. The presence of incomplete networks or dangling ends may lead to the increased dampening presented by the non-ideal network samples. It is also important to notice the decreasing width of the tan δ plots in the series, which suggest a refining of the network structure as the C_60_ fullerol concentration is increased. The initial excess of NCO to OH functional groups in synthesis would lead to a less uniform network, where a small number of fullerene urethane network bonds are likely replaced by thiourethane linkages. Finally, differences in T_g_ values deduced from the DMA and DSC measurements for the same samples would result from the inherent differences between the two techniques and also due to using different heating ramp rates in both experiments (2 °C/min in the DMA *versus* 5 °C/min for the DSC).

**Figure 3:**
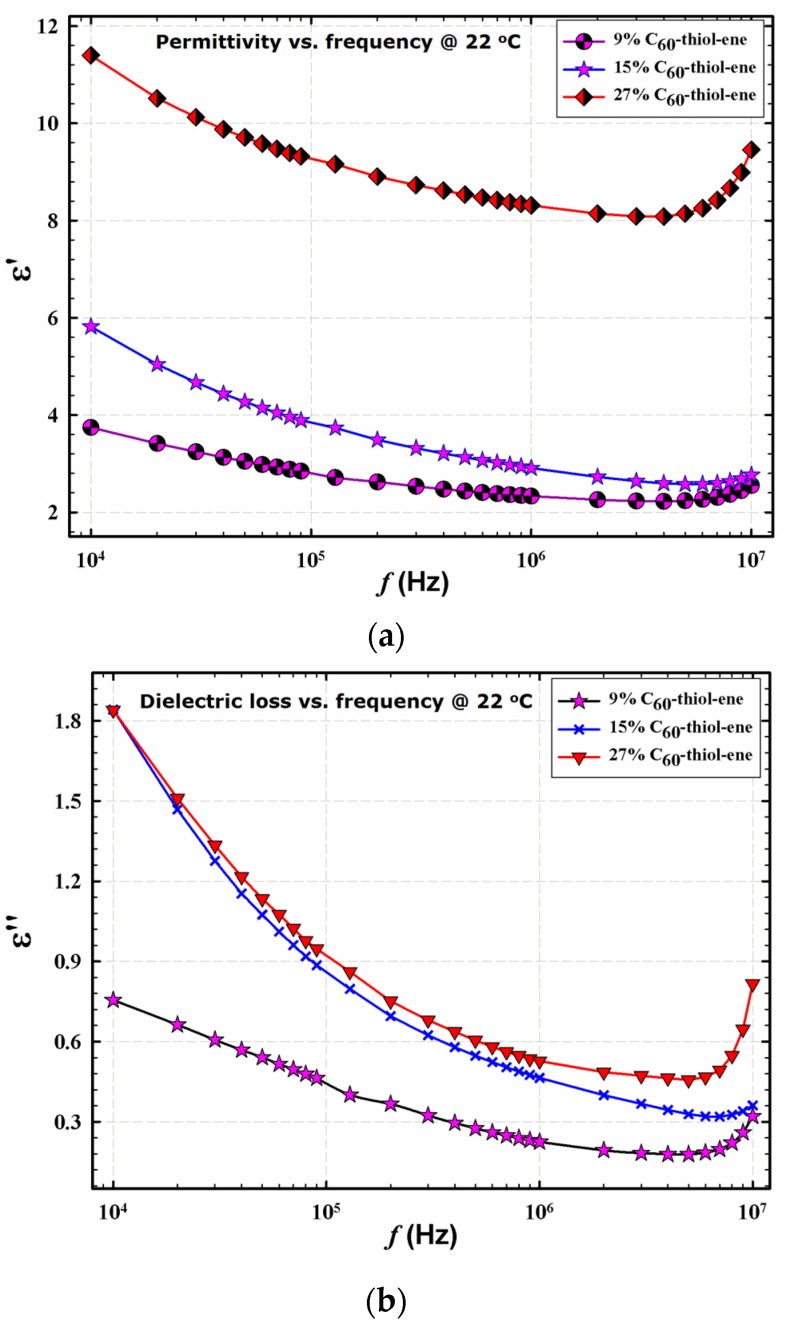
Dielectric permittivity storage (**a**); and loss (**b**) of films having different composition at 22 °C; C_60_ % noted in legend.

Figure 3 demonstrates the frequency dependences of the dielectric permittivity storage (εʹ) and loss (εʹʹ), respectively, for networks at room temperature. These spectra were measured using a Novocontrol GmbH Concept 80 broadband dielectric spectrometer. εʹ decreases with increasing frequency (*f*) and the curves are displaced upward with increasing fullerene loading. The decrease in εʹ with increasing *f* is a consequence of the fact that the time scale during which the electric field is applied in one direction; that is, one-half the period of oscillation = 1/2*f*, decreases with increasing frequency [7]. In essence, faster motions associated with polarizability have less time to be sampled. Increasing the C_60_ % serves to increase the polarizable network component. Additionally, at high fullerene loading, εʹ would increase due to higher polarizability arising from the increased number of unreacted –OH groups in the composite. Other reports have suggested that as fullerene concentration increases, the dielectric permittivity of the resultant composite films may decrease due to restriction on polymer chain motions posed by these structures, specifically polyimides containing C_60_ and C_70_ fullerenes [19] and C_60_/poly(dimethylsiloxane) [20].

**Figure 4:**
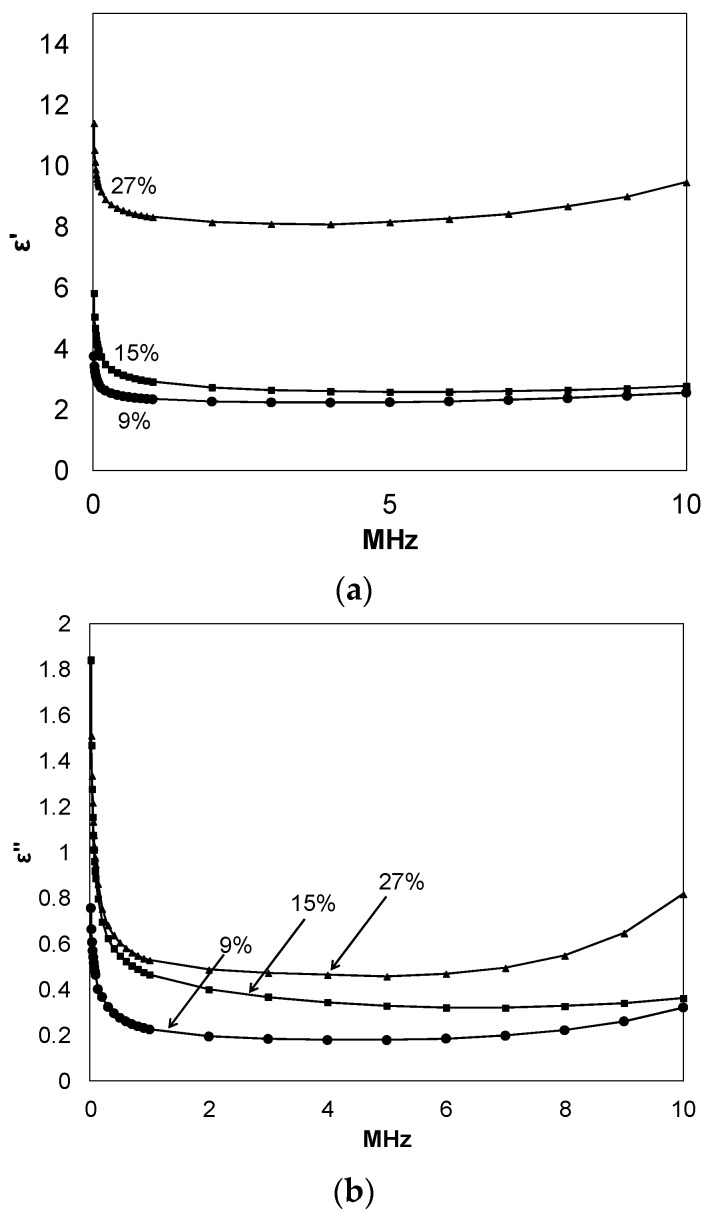
Dielectric permittivity storage (**a**); and loss (**b**) of films having different composition at room temperature, as measured by the semiconductor characterization system (SCS); C_60_% noted on plots.

εʹ and εʹʹ were also determined for the prepared thiol-ene networks using an alternate test assembly comprised of a Keithley 4200-SCS (Cleveland, OH, USA) instrument and custom electrode assembly, Figure 4. The details of this assembly are reported elsewhere [7]. When compared to the results obtained using the Novocontrol broadband dielectric spectrometer shown in Figure 3, excellent correlation exists within the same frequency range. As before, both εʹ and εʹʹ increase with increasing C_60_ loading. The initial drop in εʹ and εʹʹ values with increasing frequency are because at higher frequencies the dipoles do not have enough time to follow the alteration of the applied electric field. The increase in εʹ with frequency after 7 MHz for the 27% C_60_-thiol-ene sample might be due to a more complex polarization mechanism rather than dipole polarization. The most interesting observation in these plots is the higher values of εʹ over εʹʹ, supporting that fullerene-containing materials such as those reported herein may prove desirable for electronic and electric systems, such as in energy storage media [21,22]. Since the maximum electrical energy storage capacity (U_max_) of a linear dielectric material is given by U_max_ = εʹ·E_b_^2^/2 where E_b_ is the dielectric breakdown strength (DBS), both large εʹ and high DBS are required for large electric energy storage [22]. Consequently, incorporating materials with large εʹ within polymeric matrices of high DBS and possessing excellent mechanical properties may lead to a large energy storage dielectric material. Another indicator regarding future potential of these nanocomposites as energy storage systems can be deduced from the fact that values of the εʹ and εʹʹ can be tuned via variation of the fullerene loading. Using the laboratory methods employed herein, our group was able to produce films of high dielectric response and low loss employing only hydroxylated C_60_ as the polarizable group using a simple synthetic strategy.

## 3. Experimental Section

### 3.1. Materials

The following materials were used without further purification: C_60_ fullerene (MER, 99+%, Tuscon, AZ, USA), potassium hydroxide (Sigma-Aldrich, 85+%, St. Louis, MO, USA), Sephadex G-25 (Sigma-Aldrich, 20–80 μm bead size, St. Louis, MO, USA), allyl isocyanate (Sigma-Aldrich, 98%, St. Louis, MO, USA), dibutyltin dilaurate (DBTDL, Aldrich, 95%, St. Louis, MO, USA), trimethylolpropane tris-3-mercaptopropionate (TMPMP, Sigma-Aldrich, ≥95%, St. Louis, MO, USA), pentaerythritol allyl ether (APE, Sigma-Aldrich, 70%; remaining 30% monoene, St. Louis, MO, USA), and azobisisobutyronitrile (AIBN, Sigma-Aldrich, 98%, St. Louis, MO, USA). Chloroform (Sigma-Aldrich, 99+%, St. Louis, MO, USA) was dried over 4 Ǻ molecular sieves prior to use.

### 3.2. Preparation of Hydroxylated C_60_(OH)_x_ and C_60_-Thiol-Ene Films

The hydroxylation of C_60_ and was performed and characterized as previously reported [7]. In general, C_60_ fullerenes and KOH were combined (1:25 molar equiv.) under high-speed vibrational milling conditions using a Spex mixer/miller 8000. The resulting brown solid was dissolved in water, purified by means of Sephadex G-25 size exclusion column chromatography, concentrated, and precipitated in MeOH. Solids were collected by filtration through 0.2 μm PTFE membrane and dried under vacuum at 50 °C. The purified hydroxylated fullerenes were characterized by FT-IR spectroscopy and thermal gravimetric analysis (TGA). The dry, purified hydroxylated C_60_ was determined to have the average molecular formula C_60_(OH)_29_.

Allyl isocyanate (NCO and ene) and dry C_60_(OH)_29_ were combined at 2:1, 1:1, or 1:2 NCO/OH equivalence, where mass of C_60_(OH)_29_ was divided by total mass of nonvolatile film constituents and expressed as a percent, yielding 9, 15, and 27 wt % C_60_ films, respectively. The reaction was mixed under high shear conditions at 2500 rpm using a Speed Mixer DAC 150 FVZ-K for one hour, with the addition of DBTDL catalyst occurring after several minutes. TMPMP (SH) in dry chloroform (1 mL) was combined with the resulting ene-functionalized fullerene (1:1 SH/ene) followed by AIBN (2 wt %), with 5 min of high shear after each sequential addition. Transfers to and from reaction vessel were performed in N_2_ environment. Solvent was removed under reduced pressure and the reaction mixture was transferred to a PTFE evaporating dish for thermal curing at 65 °C. The resulting film was sonicated briefly in MeOH to quench any residual reactive NCO groups, then dried at 50 °C under reduced pressure.

### 3.3. FT-IR and Gel Fraction

The infrared spectra of the prepared samples were recorded in the wavenumber range of 400–4000 cm^−1^ using a Nicolet Nexus 470 FT-IR spectrometer (Madison, WI, USA). Gel fractions of prepared films were obtained by dissolving a known mass of film in chloroform, resting the sample for 24 h at room temperature, and recovering the insoluble mass fraction, followed by residual solvent evaporation under reduced pressure. The gel fraction is the final mass after extraction over the initial mass, expressed as a percent.

### 3.4. Thermal Gravimetric Analysis (TGA)

Thermogravimetric analysis (TGA) was used to evaluate the thermal stability of C_60_(OH)_x_ for the estimation of –OH functionalities attached to the C_60_ cage and also to evaluate the stability of the prepared fullerene-TE networks, methods as previously reported [7]. Using a TA instruments Q5000, samples were analyzed in platinum pans over the temperature range of 25–1000 °C under nitrogen and using a high-resolution heating rate of 10 °C/min. The number of hydroxyl groups attached to the fullerene cage was estimated by Equation (2):

(2) $$\frac{{\text{Formula Mass C}}_{60}}{\text{\% weight}>570{\;}^{{}^{\circ}}\text{C}}\times \frac{\text{\% weight }150-570^{\;{}^{\circ}}\text{C}}{\text{ Formula Mass}-\text{OH}}\;$$

where loss of hydroxyl addends occurs from 150 to 570 °C and the degradation of the C_60_ cage begins at >570 °C. For fullerene-TE networks, the thermal degradation onset temperature was reported as the temperature corresponding to 10% mass loss.

### 3.5. Differential Scanning Calorimetry (DSC)

TA instruments modulated differential scanning calorimetry (DSC) Q2000 instrument was used to determine the glass transition temperature of fullerene-TE films over the temperature range of −50 to 150 °C in a heat/cool/heat cycle at 5 °C/min under nitrogen. T_g_ information was obtained from the second heat cycle.

### 3.6. Dielectric Measurements

Dielectric measurements on C60-thiol-ene composites were performed using a Novocontrol GmbH Concept 80 broadband dielectric spectrometer, and data were collected over the frequency range 0.1 Hz–3 MHz at fixed temperatures in the range of −150 to 180 °C. The temperature stability of the instrument was within ±0.2 °C. Samples were kept in a humidity control chamber (Model 503-20, Electro-tech Systems, Inc., Glenside, PA, USA) with RH < 0.5% at room temperature for more than one week prior to analysis. Sample discs of 2 cm diameter that were covered with two very clean aluminum sheets on both sides were sandwiched between two gold-coated copper electrodes of 2 cm diameter and transferred to the instrument for data collection.

Dielectric properties were also measured using a Keithley 4200 semiconductor characterization system (SCS) connected to a custom designed parallel plate electrode assembly as previously reported. Two types of permittivity tests were performed with this assembly. The first test measured capacitance at frequencies spanning a range from 10 kHz to 10 MHz in graduated logarithmic steps with 0 VDC (volts dc) bias. For the second test, the DC offset voltage was swept from −30 to +30 VDC in 1 VDC steps at a constant frequency. Both tests calculated permittivity by measuring the root mean squared (rms) current at the given frequency and rms voltage. All tests were performed at room temperature (22 °C) under controlled humidity. The standard equations for parallel plate capacitor geometry were used to calculate permittivity from the measured capacitance.

### 3.7. Dynamic Mechanical Analysis (DMA)

The dynamic storage modulus (E′) as well as tan δ = Eʹʹ/Eʹ were measured using a DMA Thermal Analysis Q800 instrument. All samples were run in tensile mode with a frequency of 1 Hz and amplitude of 15 μm. The experiments were performed over the temperature range −150 to 100 °C at a heating rate of 2 °C/min.

## 4. Conclusions

Alkyl thiols and ene-functionalized C_60_ fullerene were thermally polymerized to form C_60_ fullerene-thiol-ene networks. Physical and electrical properties of these networks were studied as a function of the C_60_ content. Gel fractions of the resulting composite networks are high, ranging from 88%–100% and thermal stabilities do not vary significantly with composition. DMA and DSC analyses of the thiol-ene networks illustrate a tunable T_g_ which decreases with increasing C_60_ loading and is attributed to an increase in network free volume. A sub-T_g_ transition in the range of −90 to −50 °C is ascribed to local crankshaft motions in the methylene sections of the chains. The broadband dielectric spectrometer and the Keithley SCS measurements of the networks revealed higher εʹ values as fullerene concentration increases. This was attributed to the increase in the concentration of the more polarizable network component and the freely rotating cage-surface hydroxyls. Along with increasing the dielectric permittivity, increasing the hydroxylated fullerene loading relative to other film constituents has led to a refining of the network structure as evidenced by a narrower glass transition and extremely high gel fraction for this composition. A general conclusion that can be drawn from these overall results is that this class of materials can be rendered quite polarizable, and the fact that εʹ is considerably greater than εʹʹ suggests that they may prove useful as high-energy dielectric storage media for high capacitance applications.
